# Reduced Grid Cell‐Like fMRI Activity relates to Synaptic Biomarkers in Predementia Alzheimer's Disease

**DOI:** 10.1002/alz70856_107406

**Published:** 2026-01-11

**Authors:** Jonas Alexander Jarholm, Marcia Bécu, Gøril Rolfseng Grøntvedt, Ivan Krasovec, Per Selnes, Sigrid Botne Sando, Ann Brinkmalm, Johanna Nilsson, Tobias Navarro Schröder, Atle Bjornerud, Tormod Fladby, Kaj Blennow, Tora Bonnevie, Christian F. Doeller

**Affiliations:** ^1^ Institute of Clinical Medicine, University of Oslo, Oslo, Norway; ^2^ Akershus University Hospital, Nordbyhagen, Norway; ^3^ Kavli Institute for Systems Neuroscience, Norwegian University of Science and Technology, Trondheim, Norway; ^4^ University Hospital of Trondheim, Trondheim, Norway; ^5^ K.G. Jebsen Centre for Alzheimer's Disease, Norwegian University of Science and Technology, Trondheim, Norway; ^6^ Norwegian University of Science and Technology, Trondheim, Norway; ^7^ Clinical Neurochemistry Laboratory, Sahlgrenska University Hospital, Mölndal, Sweden; ^8^ Institute of Neuroscience and Physiology, The Sahlgrenska Academy at the University of Gothenburg, Mölndal, Sweden; ^9^ Department of Physics, University of Oslo, Oslo, Oslo, Norway; ^10^ Computational Radiology and Artificial Intelligence (CRAI) Research Group, Division of Radiology and Nuclear Medicine, Oslo University Hospital, Oslo, Norway; ^11^ Akershus University Hospital, Nordbyhagen, Norway, Norway; ^12^ Max Planck Institute for Human Cognitive and Brain Sciences, Leipzig, Germany

## Abstract

**Background:**

The Entorhinal cortex (EC) is affected early by Alzheimer's disease (AD) pathology and is the main anatomical location of grid cells. Our aim was to determine whether grid‐cell‐like activity, and hippocampal task‐related activity, as measured by blood oxygen level‐dependent (BOLD) functional magnetic resonance imaging (fMRI), was associated with AD disease progression and specific biomarkers.

**Method:**

We invited participants to perform a virtual reality (VR) navigation task during fMRI. Participants were stratified as amyloid positive (+) or negative (‐) by CSF amyloid‐beta (Aß) 42/40‐ratio, and classified as cognitively normal (CN) or mild cognitive impairment (MCI) based on a standardized cognitive test‐battery. Controls were CN Aß‐ (*n* = 20), while cases were CN Aß+ (*n* = 21) and MCI Aß+(*n* = 14).«Gridness» was defined as the strength of the hexadirectional modulation of the BOLD signal in EC, observed during virtual environment navigation as participants memorized and retrieved 4 objects in a 3T scanner. We used a general linear model to test the relationship between the fMRI parameters (gridness and hippocampal task‐related activity) and the following biomarkers: cerebrospinal fluid (CSF) phosphorylated tau (ptau) 181, plasma ptau217, and CSF 14‐3‐3 zetadelta, a novel biomarker of synaptic degradation.

**Result:**

Significant gridness was only found in controls, not in cases. Compared to controls, there was significantly lower gridness in MCI Aß+ (*p* <0.01) but only trending towards lower gridness (*p* = 0.078) in CN Aß+. Hippocampal task‐related activity was significantly higher in both CN Aß+ (*p* <0.05) and MCI Aß+ (*p* <0.001) compared to controls. The association between gridness and biomarkers was trending for CSF ptau181 (*p* = 0.064), significant for plasma ptau217 (*p* = 0.05), and highly significant for CSF 14‐3‐3 zetadelta (*p* <0.01). The association with hippocampal task‐related activity was not significant for CSF ptau181, and trending for plasma ptau217 (*p* = 0.057) and CSF 14‐3‐3 zetadelta (*p* = 0.054).

**Conclusion:**

Lower gridness and higher hippocampal task‐related activity was associated with more advanced predementia AD, also before cognitive impairment or MTL atrophy. Lower gridness could be caused by altered neuronal activity and computation. Gridness had the strongest association to biomarkers, especially CSF 14‐3‐3 zetadelta, suggesting that synaptic degradation could be an important hallmark of grid cell dysfunction in AD.

